# Association Between Three-Dimensional Transrectal Ultrasound Findings and Tumor Response to Neoadjuvant Chemoradiotherapy in Locally Advanced Rectal Cancer: An Observational Study

**DOI:** 10.3389/fonc.2021.648839

**Published:** 2021-06-04

**Authors:** Xun Zhang, Jin Fan, Lijie Zhang, Jingwen Wang, Minghe Wang, Ji Zhu

**Affiliations:** ^1^ Department of Ultrasound Diagnosis, Fudan University Shanghai Cancer Center, Shanghai, China; ^2^ Department of Radiation Oncology, Fudan University Shanghai Cancer Center, Shanghai, China; ^3^ Department of Oncology, Shanghai Medical College, Fudan University, Shanghai, China; ^4^ Department of Colorectal Surgery, Fudan University Shanghai Cancer Center, Shanghai, China; ^5^ Department of Abdominal Radiation Oncology, The Cancer Hospital of the University of Chinese Academy of Sciences (Zhejiang Cancer Hospital), Institute of Basic Medicine and Cancer (IBMC), Chinese Academy of Sciences, Hangzhou, China; ^6^ Zhejiang Key Laboratory of Radiation Oncology, Hangzhou, China

**Keywords:** three-dimensional, ultrasound, neoadjuvant chemoradiotherapy, rectal cancer, complete response (CR)

## Abstract

**Background:**

There is a significant demand for the development of non-surgical methods for the evaluation of complete response to tumor therapy. Predicting ability and image quality of routine imaging has not been satisfactory. To avoid the deficiencies, we assessed the capability of three-dimensional transrectal ultrasound in predicting the response to neoadjuvant chemoradiotherapy in rectal cancer patients.

**Methods:**

The inclusion criteria were patients with locally advanced rectal adenocarcinoma, receiving capecitabine-based neoadjuvant chemoradiotherapy, distance from anal verge (≤6 cm), clinical stage T3-4 and/or N+ without evidence of distant metastasis, and restaging ycT0-3a (T3a <5 mm) after the end of neoadjuvant chemoradiotherapy. Three-dimensional transrectal ultrasound was performed 7 weeks after neoadjuvant chemoradiotherapy to discern the patients with complete response from the others. Eight main parameters were obtained from three-dimensional transrectal ultrasound: thickness of muscularis on the residual side, thickness of contralateral muscularis, angle of residual arc, regularity of the shape, integrity of the mucosal layer, blurring of the margin, internal echo, and posterior echo. The association between tumor response and three-dimensional transrectal ultrasound parameters was analyzed, and a model was developed by logistic regression.

**Results:**

Between 2014 and 2019, 101 patients were recruited; 72 cases received total mesorectal excision, and 29 cases underwent watch-and-wait. Among the three-dimensional transrectal ultrasound parameters, the adjusted-thickness of the muscularis (*P*<0.01), angle of the residual arc (*P*<0.01), and regularity of the residual shape (*P*<0.01) were strongly associated with tumor response. In the dataset with total mesorectal excision cases (TME dataset), the residual adjusted-thickness (odds ratio [OR]=4.88, 95% confidence interval [CI]=1.44–16.6, *P*=0.01) and regularity of the residual shape (OR=5.00, 95% CI=1.13–22.2, *P*=0.03) were kept in the final logistic model. The area under the curve of the logistic model was 0.84. Among these parameters, residual adjusted-thickness correlated significantly with tumor response. Additionally, we observed similar results in the whole population of 101 cases (whole dataset) and in the cross-validation.

**Conclusion:**

Three-dimensional transrectal ultrasound model is a valuable method for predicting tumor response in rectal cancer patients undergoing neoadjuvant chemoradiotherapy, which should be included as a factor for evaluating clinical complete response.

**Trial Registration:**

This trial was registered with ClinicalTrials.gov, number NCT02605265. Registered 9 November 2015 - Retrospectively registered, https://clinicaltrials.gov/ct2/show/record/NCT02605265

## Introduction

For locally advanced rectal cancer (LARC) patients, neoadjuvant chemoradiotherapy (NACRT) followed by total mesorectal excision (TME) is the standard treatment (1). NACRT had a good downstaging effect on primary tumor and approximately 10%–30% of patients showed pathologically complete response (2, 3). Before surgery, clinical tumor response to NACRT is evaluated by lesion morphology using imaging techniques, such as computed tomography (CT), magnetic resonance imaging (MRI), and positron emission tomography-CT. However, the accuracy of evaluating complete response with non-surgical methods is limited and a more powerful method is needed.

Endoscopic ultrasonography (EUS) showed a potential predictive ability to detect tumor regression grade 3–4 (TRG 3–4, >10% vital tumor cells) residual disease, 12 weeks after completion of NACRT with a sensitivity of almost 90% in esophageal cancer. It could also detect cases without vital tumor cells with the sensitivity of >50% (4). Another study proved that sequential EUS examination might predict the therapeutic efficacy of preoperative CRT for LARC (5). The T stage detected by three-dimensional (3D) reconstruction was more accurate than conventional techniques (EUS), as the structure of the rectal wall was clearer, and the image could be viewed from different angles through 3D reconstruction (6). The accuracy of 3D-TRUS for the assessment of infiltration depth was 88%, compared with 82% for EUS (7). A similar study compared 3D-TRUS and EUS for rectal cancer staging and found 91% accuracy of 3D-TRUS for pT2 and 85% for pT3 stages, whereas the accuracy of EUS for the two stages was 85% and 76%, respectively (8).

Therefore, the purpose of this study was to assess the capability of 3D-TRUS in assessing the response of locally advanced rectal cancer patients to preoperative CRT.

## Materials and Methods

### Patients and Design

**Figure 1:**
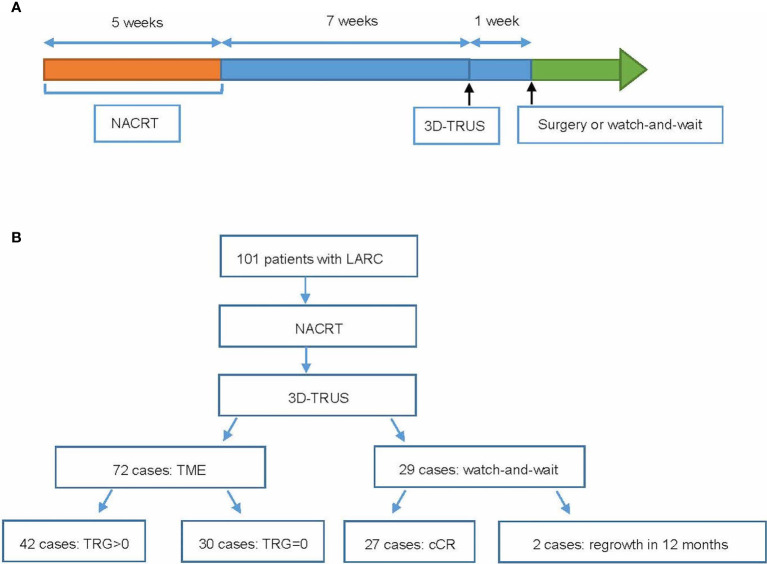
Study design and recruitment. **(A)** Study design. **(B)** Recruitment overview.

Patients with locally advanced rectal adenocarcinoma were enrolled at Fudan University Shanghai Cancer Center. The inclusion criteria were receiving NACRT (intensity-modulated radiation therapy of 50 Gy in 25 fractions concurrently with capecitabine-based monotherapy or capecitabine plus irinotecan), the distance from anal verge (≤6 cm) not suitable for surgery to preserve anus, and restaging ycT0-3aN0 by pelvic MRI after the end of NACRT according to the Radiologic Society of North America criteria (T3a <5 mm) (**Figure 1**) (9). Patients who met the inclusion criteria in our previous trials; Expansion and CinClare were included in this study (10–12). Details of NACRT and consolidation chemotherapy have been described in our previous manuscript (10–12).

After the completion of NACRT, patients were restaged by pelvic MRI, and only patients judged as ycT0-3aN0 would take 3D-TRUS. 3D-TRUS performed about 7 weeks after NACRT aimed to discern patients with complete response (CR) from those without. According to the initial therapeutic scheme, all patients were scheduled to undergo a radical surgery 8 weeks after CRT. However, because over 30% of the patients in the experimental group achieved pathological complete response (pCR) in our previous CinClare trial (10), an increasing number of patients were willing to preserve their anal function after obtaining a good tumor response after NACRT. Therefore, the therapeutic scheme was modified such that either TME or watch-and-wait were options, while considering the consent of the patients and the evaluation of tumor response by imaging and clinical examinations. The median time for local regrowth was 12 months, indicating that 50% or more of local regrowth would occur within 12 months (13, 14). For patients who maintained cCR during the 2-year observation, shortening the period could reduce their mental stress and improve the quality of life. Based on the above considerations, the clinical complete response (cCR) was defined as complete response without tumor regrowing during close follow-up of >12 months. Watch-and-wait meant that radical surgery was withheld and replaced with close observation and follow-ups in patients who achieved a cCR, evaluated by digital rectal exam, pelvic MRI, and endoscopy according to Memorial Sloan Kettering Cancer Centre criteria (15).

### Pathological Evaluation of Tumor Response

Pathological tumor response was evaluated according to the 2010 American Joint Committee on Cancer TRG system (16). Details of the AJCC TRG system were defined as follows: TRG 0, defined as no viable cancer cells; TRG 1, characterized by a single or small groups of tumor cells; TRG 2, involved residual cancer outgrown by fibrosis, but fibrosis still predominating; and TRG 3, defined as minimal or no tumor cells killed. To establish the model for predicting pathological response, we divided the surgical population into two groups of TRG=0 or not. Data of patients subjected to watch-and-wait were included in a sensitivity test. As the cases without tumor regrowth in 12 months could be regarded as cCR, we divided the whole dataset into two groups, which were as follows: (1) TME patients with TRG=0 or patients who achieved cCR; and (2) TME patients with TRG>0 or watch-and-wait patients with tumor regrowth.

### Three-Dimensional Transrectal Ultrasound

Ultrasonographic examination was performed on Danish BK Company Flex Focus 1202 ultrasound scanner with 10 cm effective length rectum 3D probe (frequency 8-15 HZ). Before the test, 60 ml of enema was administered into the anus of patients, 1 h in advance to try to empty stool. The surface cover of the ultrasound probe was a special layer of probe cover, and the surface of the probe cover was coated with a coupling agent. Patients lay on their left side, their knee joint was bent as close to their chest as possible, and the anus was exposed. Before the examination, an anal digital examination was performed to have a preliminary understanding of the approximate location and texture of the tumor. The ultrasound probe was then slowly inserted in-sync with the patient’s breathing. In the anus, to reduce the discomfort of the patient, the ultrasound probe was gently inserted into the rectum until it passed through the lesion, to observe the entire picture of the tumor. At the same time, 50–100 ml of normal saline was injected for the detection and dynamic observation of the infiltration depth in the intestinal wall and the metastasis to lymph nodes around the lesion. There were eight main parameters obtained from 3D-TRUS, which were as follows: thickness of muscularis on the residual side, thickness of contralateral muscularis, angle of residual arc, regularity of the shape, integrity of the mucosal layer, blurring of the margin, internal echo, and posterior echo (**Table 1**).

**Table 1:** Definition of 3D-TRUS parameters.

Parameter name	Definition of parameter
**Thickness of muscularis on the residual side**	Thickness of muscularis on the residual side or the maximum thickness of the muscularis around the residual
**Thickness of contralateral muscularis**	Thickness of contralateral muscularis or the thickness of the muscularis on the opposite side of residual
**Angle of residual arc**	Angle of residual arc or the angle of arc around the bowel wall
**Regularity of shape**	Regularity of the shape or the regular or irregular shape of residual
**Integrity of mucous layer**	Mucosal layer around the residual was intact, thinned, or interrupted
**Blurring of margin**	Blurred or clear margin of the residual
**Internal echo**	Echo inside the residual was homogeneous or heterogeneous
**Posterior echo**	Echo behind the residual was normal or weakened

The side with residual tumor was defined as residual side. Thickness of the muscularis on the residual side was defined as the maximum thickness of the muscularis around the residual side. Thickness of contralateral muscularis was defined as the thickness of the muscularis opposite to the residual side. Angle of residual arc was the angle of arc around the bowel wall. In detail, the angle between the two lines connecting two ends of the residual tumor to the center point of the rectal cross-section was the “angle of residual arc”. Regularity of the shape referred to the regular or irregular shape of the residual tumor. Integrity of the mucosal layer was described as either intact, thin, or interrupted mucosal layer around the residual tumor. The blurring of the margin referred to the presence of blurred or clear margins of the residual tumor. Internal echo referred to the homogeneous or heterogeneous echo inside the residual tumor. The posterior echo referred to the echo on the posterior side the residual tumor, which was either normal or weakened. All the ultrasound parameters used in this study took the same standards in TI-RADS and BI-RADS (17, 18).

The diagnosis of 3D-TRUS in our center was managed by ultrasound specialists who were blinded to treatment details. The clinical doctors evaluated cCR status by 3D-TRUS results and other information, and compared the conclusions with surgical pathology results. The final report of 3D-TRUS was confirmed and issued by two ultrasound specialists and the standards were unified through training.

### Statistical Analyses

The statistical analyses were performed by R, version 3.5.1. To better evaluate the tumor response (TME dataset: TRG=0 *vs.* TRG>0) after NACRT, a logistic regression model was built. The patients who underwent watch-and-wait were not included in the training stage, as the pathological evaluation was unavailable for them. Therefore, these cases could be included to validate the model (sensitivity analysis in whole population). The stepwise regression, forward method was used to select parameters to build the final logistic model, with which *P*-value <0.05 was kept in the final model. The area under the receiver-operating characteristic curve (AUC) was calculated to evaluate the discrimination of the model, and 1000 times bootstrap sampling was used to calculate the corresponding confidence interval (CI). By 1000 times three-fold cross-validation, the performance of the model was validated, and it was also validated in the whole dataset (response variable: TRG=0 or without regrowth *vs.* TRG>0 or with regrowth).

## Results

### Baseline Characteristics

A total of 101 patients were recruited between November 2014 and June 2019, and the follow-up time was >12 months for all patients. Eight patients came from Expansion trial, 18 patients came from CinClare trial, and 75 patients were recruited only in this trial. Among them, 72 patients received TME, and 29 patients underwent watch-and-wait. The median [interquartile range (IQR)] age was 56 [49–62] years, and 62/101 (61%) were male. The median [IQR] duration between the completion of NACRT and 3D-TRUS was 7 [6–9] weeks. The median [IQR] interval between the completion of NACRT and surgery was 8 [7–10] weeks. All patients received concurrent chemotherapy, including capecitabine alone (36.1%) or capecitabine plus irinotecan (63.9%). Detailed clinical characteristics of the participants are shown in **Table 2**. Postoperative pathological examination was performed on all of the surgical specimens. In the patients who underwent watch-and-wait, 2 (6.9%) relapsed within 12 months, and the other 27 patients (93.1%) maintained a cCR status for a median of 32 [24–38] months.

**Table 2:** Baseline characteristics of the patients with TRG = 0, TRG > 0, and watch-and-wait group.

	TRG = 0	TRG > 0	Watch-and-wait
**Total, No.**	30	42	29
**Sex, No. (%)**			
**Female**	13 (43.3)	21 (50)	5 (17.2)
**Male**	17 (56.7)	21 (50)	24 (82.8)
**Age, median (IQR)**	56 (47-59)	56 (48-62)	58 (52-64)
**Clinical T stage, No. (%)**			
**T2**	3 (10)	0 (0)	10 (34.5)
**T3**	23 (76.7)	38 (90.5)	18 (62.1)
**T4**	4 (13.3)	4 (9.5)	1 (3.4)
**Clinical N stage, No. (%)**			
**N0**	3 (10)	0 (0)	5 (17.2)
**N1**	15 (50)	21 (50)	12 (41.4)
**N2**	12 (40)	21 (50)	12 (41.4)
**Baseline Tumor Max Length, median (IQR), cm**	4.8 (3.35-5.90)	5.15 (4.03-5.95)	4.7 (3.28-5.87)
**Baseline CEA, median (IQR), ng/mL**	3.76 (1.08-6.17)	4.21 (1.56-13.21)	3.58 (1.12-6.03)

TRG, tumor regression grade; IQR, interquartile range; CEA, carcinoembryonic antigen.

### Univariate Analysis

**Figure 2:**
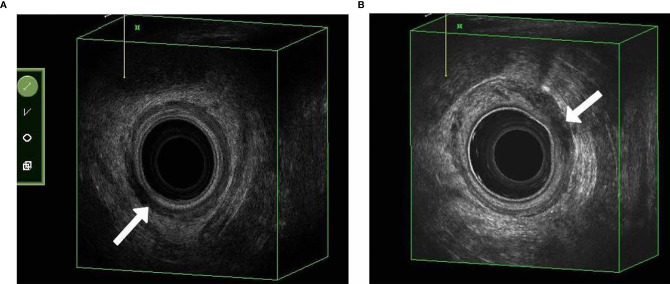
3D-TRUS pictures. **(A)** A case with complete response determined by three-dimensional transrectal ultrasound (3D-TRUS) and confirmed by TME pathological report (TRG = 0). **(B)** A case with tumor residue which was also confirmed by TME pathological report with TRG = 3.

**Figure 3:**
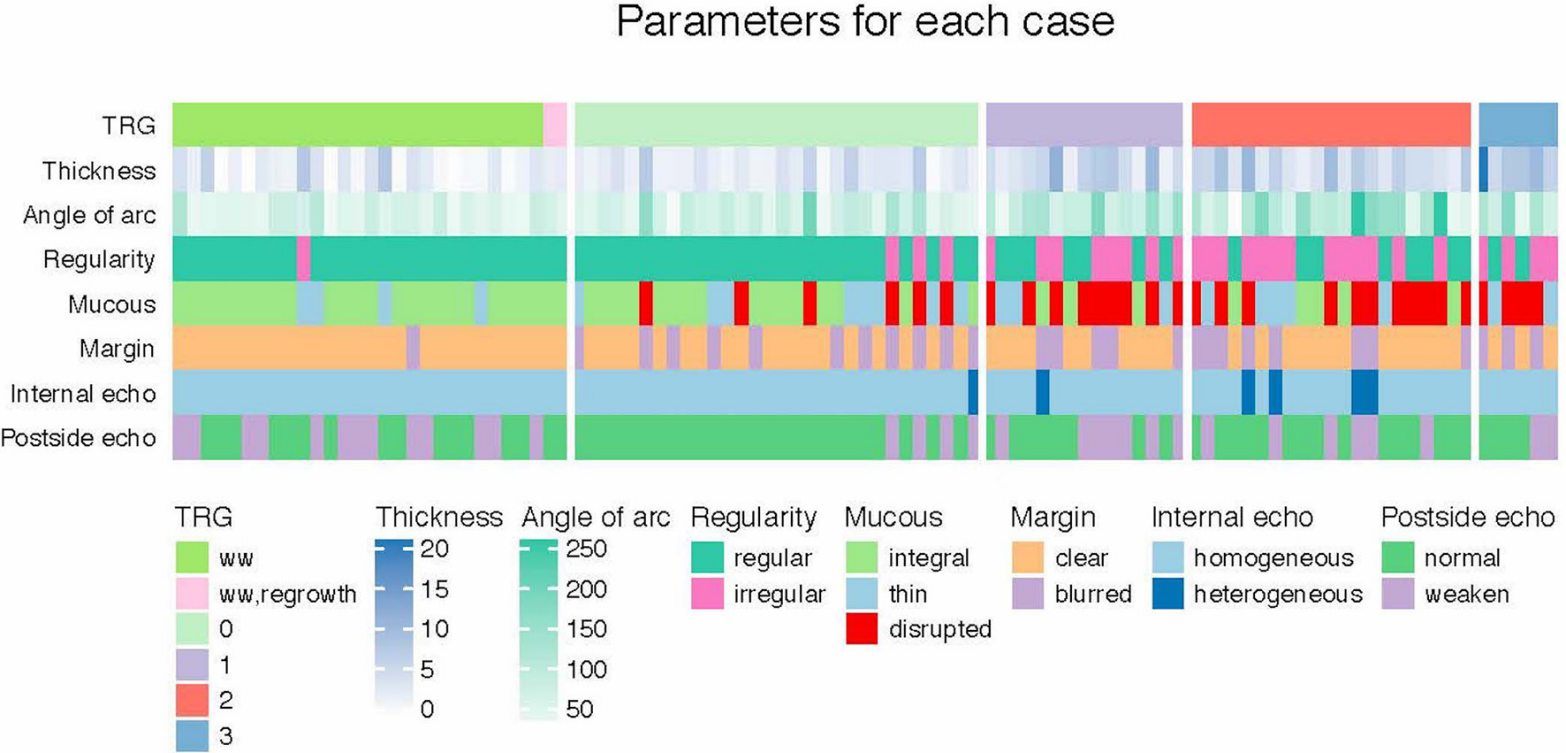
Data distributions of the main three-dimensional transrectal ultrasound (3D-TRUS) parameters.

The snapshots of 3D-TRUS images were shown in **Figure 2**. The adjusted-thickness was calculated (adjusted-thickness = thickness of muscularis on the residual side – thickness of contralateral muscularis). Data distributions of the parameters were shown in **Figure 3**. The association between tumor response and 3D-TRUS parameters was analyzed. Firstly, we focused on the dataset of 72 cases with TME (TME dataset), as the pathological examination was the golden standard to evaluate the residual tumor. Univariate analysis showed that adjusted-thickness (*P*<0.01), angle of residual arc (*P*<0.01) and regularity of the shape (*P*<0.01) were strongly associated with pathological response (TRG=0 or TRG>0).

**Figure 4:**
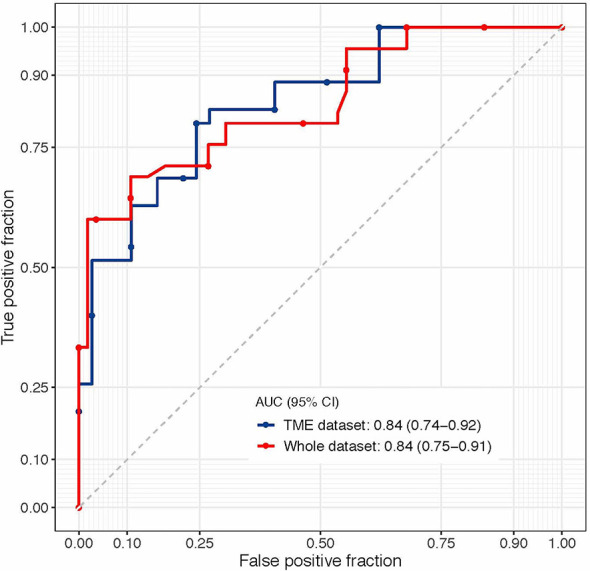
The ROC of the final model (incorporating adjusted-thickness and regularity of the shape).

To predict the tumor response more accurately, we developed a logistic model with the stepwise forward method in the TME dataset. In the final 3D-TRUS model, the following two variables were retained: adjusted-thickness (odds ratio [OR]=4.88, 95% CI=1.44–16.6, *P*=0.01) and regularity of the shape (OR=5.00, 95% CI=1.13–22.2, *P*=0.03); the AUC of the model was 0.840 (95% CI=0.739–0.920; **Figure 4**). Cross-validation of the model was performed in 1000 times three-fold, and the average AUC of the model was 0.836.

The formula of the model was as follows: logit(y) = 1.585 × adjusted-thickness + 1.609 × regularity of the shape. Adjusted-thickness was the continuous variable, and regularity of the sharp was the categorical variable (regular=0, irregular=1). The smaller the y value, the greater the probability of pCR. When y value equaled to 4.582, the value of Youden index reached maximum; thus, the cut-off point to predict pCR was 4.582.

To validate the model in the whole population, we performed a sensitivity analysis in the whole dataset (101 cases), and we obtained similar results: the AUC was 0.841 (95% CI=0.752–0.910) for this final 3D-TRUS model (incorporating adjusted-thickness and regularity of the shape). The predicting results of watch-and-wait patients by the model was showed in **Table S1**.

### Residual Adjusted-Thickness

It is worth noting that the adjusted-thickness was the most powerful predictor (TME dataset: AUC=0.817; whole dataset: AUC=0.811). We, therefore, focused on this parameter. By calculating the maximum value of the Youden index in the TME dataset, we determined that the optimal cut-off value for the adjusted-thickness was 3.55 mm, which correctly detected the TRG 0 cases with a sensitivity of 73%, a specificity of 81%, and a classification accuracy of 78%.

## Discussion

The current study demonstrates that the measurements of 3D-TRUS at 7 weeks after completion of NACRT were significantly associated with tumor response in patients treated with curative intent for rectal cancer and reached ycT0-3aN0 evaluated by pelvic MRI. We established a model based on adjusted-thickness and regularity of the shape (AUC=0.84) to predict pCR. These results were robust in cross-validation and in the sensitivity analysis, indicating that 3D-TRUS would be an excellent method for evaluating the residual tumor. The adjusted-thickness assessed by 3D-TRUS was especially noteworthy.

According to the literature review, surgical mortality in advanced rectal cancer is approximately 2% to 8% (19), and nearly half of the patients have long-term complications, including intestinal obstruction, urinary incontinence, and sexual dysfunction (20). NACRT had a good downstaging effect in some selective patients, without any residual tumor (3). In our center, irinotecan-based chemoradiotherapy has been used in a series of clinical trials (10, 11). Because of the excellent result of CinClare trial, irinotecan-based CRT was listed as an option in Chinese CSCO colorectal cancer guideline. The need for radical surgery in patients who reached CR was challenged. To avoid this unnecessary radical surgery, a new strategy called watch-and-wait was introduced. It meant that radical surgery was excluded in patients who achieved a cCR and was replaced by close observation and follow-up (12). The value of this strategy was to preserve the patients’ anal function and to improve their quality of life without shortening overall survival. In these trials, 3D-TRUS was used to evaluated tumor response as a new technology. We hope it can potential improve the accuracy of complete response for watch and wait approach.

The cCR referred to the complete remission of tumor, determined by clinical and imaging examinations. A criteria for evaluating cCR was proposed. Generally, MRI, endoscopy, and digital rectum examination were the main approaches to assessing tumor response. MRI was widely used in evaluating tumor stage, depth of tumor invasion, invasion of adjacent tissue, and the involvement of lymph nodes. Compared with the final pathological stage, the sensitivity and specificity of MRI in assessing tumor response were 67% and 95%, respectively (21). Since MRI lacked sensitivity for assessing tumor response, it alone might not be suitable for predicting pCR. EUS was also paid close attention by many researchers. However, studies showed that the accuracy of EUS in evaluating tumor regression was low (38% to 75%) (22). Recently, Nahas et al. summarized several similar trials and found that some patients who did not meet the cCR criteria were confirmed by postoperative pathology to have reached pCR (21). The main reason for this difference was that, even if some patients reached pCR, their mucosa still showed small ulcers and stiffness. Fibrosis in some areas could not be distinguished from the tumors, and accurate judgment could not be made through clinical and imaging examinations (21). Hiotis et al. also found that only 25% of patients with cCR reached pCR, and the remaining 75% of patients with cCR had tumor residues (23). Therefore, it is critical to follow the watch-and-wait approach to unravel those real pCR cases. In this study, we found out that 3D ultrasound had relatively higher sensitivity and specificity, and it should be listed as an important criterion for evaluating cCR in clinical practice.

With the advent of 3D ultrasound in the rectum, this technology has been applied to the clinical setting for >20 years since the 1990s (24). In tumor staging, the specific data derived from the 3D reconstruction for the assessment of T invasion and nodal involvement was more accurate than those from conventional techniques (EUS and CT). Compared with traditional two-dimensional ultrasound, the image quality was also better, and the frequency was higher. The anatomical relationship between the rectum and surrounding tissues and organs was more clearly displayed as a five-layered rectal wall. In addition, the image could be viewed from different angles, which was convenient for preservation and repeated observations, and overcame the weakness of two-dimensional ultrasound of poor repeatability (25). It was reported in the literature that rectal 3D-TRUS had advantages over traditional two-dimensional ultrasound in determining the T and N stages of tumors, and the accuracy was higher, reaching 72%–95% (26–29). In the present study, the accuracy of 3D-TRUS in determining the depth of rectal cancer invasion was 91.6%, which was consistent with the current literature. Taking the 3D-TRUS method as a part of the criteria of cCR would significantly improve the accuracy of the evaluation. The significance of this study was to explore the value of 3D-ultrasound to improve the accuracy of comprehensive clinical assessment.

There were some limitations to this study. First, the quality of ultrasound parameters needed strengthening and the model lacked an external test. It was difficult to control the quality of 3D-ultrasound examinations in other hospitals, so it was difficult to conduct external verification at this stage. We will verify this model in subsequent clinical studies and conduct further research. There was more information to be excavated, such as using ultrasonic imaging omics to extract higher order features and more research was necessary to ensure that the model was robust and reproducible. Second, lymph node detection of 3D-TRUS was not satisfactory, and single examination might not be enough for the prediction of pCR. Considering the desire of preserving organ function and importance of T stage in recurrence and regrowth, the advantage of 3D-TRUS in evaluating T stage is even more critical in clinical practice (Local Recurrence After Complete Clinical Response and Watch and Wait in Rectal Cancer After Neoadjuvant Chemoradiation: Impact of Salvage Therapy on Local Disease Control). However, the depth of the ultrasound probe was enough, as the watch-and-wait strategy was recommended for low rectal cancer. Third, the model only included ultrasound parameters in the model, but not clinical indicators. After statistical calculations, the clinical indicators collected in this experiment were not suitable for joining this model, and could not improve the predictive ability of the model. We will collect other clinical indicators in a targeted manner in subsequent studies and try to build more complex models. Lastly, we did not perform the 3D-TRUS at baseline that could be matched in pairs with post-NACRT 3D-TRUS to adjust the parameters. We have initiated an expanded cohort to further validate this research and would collect baseline and post-NACRT 3D-TRUS parameters in pairs.

This study showed a pathological tumor response predicting model, adequately based on residual size, angle of lesion arc, and the regularity of lesion shape, could predict tumor response with AUC of 0.89 in rectal cancer patients who completed NACRT. Therefore, these 3D-TRUS-based measurements might be helpful in the follow-up of rectal cancer after completion of NACRT.

## Data Availability Statement

The raw data supporting the conclusions of this article will be made available by the authors, without undue reservation.

## Ethics Statement

The studies involving human participants were reviewed and approved by Central Ethics Committee of Fudan University Shanghai Cancer Center (Shanghai, China). The patients/participants provided their written informed consent to participate in this study.

## Author Contributions

XZ performed the 3D-TRUS examinations of the rectum. JF analyzed the patient data regarding the 3D-TRUS parameters and developed a 3D-TRUS model using logistic regression. LZ was a major contributor in writing the manuscript. JW helped organize the clinical data of patients. MW and JZ designed and organized this study. All authors contributed to the article and approved the submitted version.

## Funding

This work was supported by the Natural Science Foundation of Shanghai (grant number: 19ZR1410600) and Science Popularization Project of Science and Technology Commission of Shanghai Municipality (CN) (grant number: 19DZ2304900). The funders had no role in the design and conduct of the study; collection, management, analysis, and interpretation of the data; preparation, review, or approval of the manuscript; and decision to submit the manuscript for publication.

## Conflict of Interest

The authors declare that the research was conducted in the absence of any commercial or financial relationships that could be construed as a potential conflict of interest.

The reviewer [YD] declared a shared affiliation, with no collaboration, with the authors to the handling editor at the time of the review.

## Abbreviations

3D-TRUS, three-dimensional transrectal ultrasound; AUC, area under curve; cCR, clinical complete response; CRT, chemoradiotherapy; CT, computed tomography; IQR, interquartile range; MRI, magnetic resonance imaging; NACRT, neoadjuvant chemoradiotherapy; pCR, pathological complete response; TME, total mesorectal excision; TRG, tumor regression grade.
