# Supplementary material for: Association Between Three-Dimensional Transrectal Ultrasound Findings and Tumor Response to Neoadjuvant Chemoradiotherapy in Locally Advanced Rectal Cancer: An Observational Study
Source: Front Oncol. 2021 Jun 4;11:648839. doi: 10.3389/fonc.2021.648839 (PMC8223675; doi:10.3389/fonc.2021.648839)
Supplement: Supplementary file 1 [file Table_1.pdf]

Table S1. Predicting results and follow-up results of watch-and-wait patients.

**Watch-and-wait patients**	**cCR > 12 months (n = 27)**	**Regrowth in 12 months (n = 2)**	**Total (n = 29)**
**Predicting model determined cCR**	**24**	**2**	**26**
**Predicting model determined non-cCR**	**3**	**0**	**3**
